# The Chancellor of the Exchequer's Lack of Statesmanship

**Published:** 1912-07-13

**Authors:** 


### The Hospital

The Workers' Newspaper of Administrative Medicine and Institutional Life, Administration, National Insurance and Health.

No. 1358, Vol. LII. SATURDAY, JULY 13, 1912. PRICE ONE PENNY.

### THE CHANCELLOR OF THE EXCHEQUER'S LACK OF STATESMANSHIP.

For the first time in English history the British nation has before it to-day the amazing spectacle of a Chancellor of the Exchequer who, despite many considerable gifts, lacks statesmanship, though he seems incapable of recognising this painful truth. Our readers are fully aware, from our constant support and criticism of the Bill itself before it became an Act and of the National Insurance Act, that we, in common with the majority of the inhabitants of Great Britain, quite apart from politics, are strong supporters of and believers in a statesmanlike measure of national insurance. When Mr. Lloyd George took up national insurance the whole country, irrespective of party, welcomed his intervention and looked to him with hope for a wise solution of a difficult problem. When the Bill was introduced, in the belief which legislative precedent justified that Mr. Lloyd George wished to be first of all a statesman and not a party man where national insurance was concerned, all parties in the House of Commons welcomed the Chancellor of the Exchequer's proposals. Mr. Lloyd George lost no opportunity at the beginning in asking for the co-operation of everybody to make national insurance a success, and to place it on the wisest and most practical and workmanlike lines. Had he maintained the position with which he started the country might to-day have been blessed by an Act of national insurance the advent of which would have been welcomed by the whole community.

Mr. Lloyd George's instincts were sound enough; his method was to call in consultation the men who he was advised were the most capable and knowledgeable in regard to the many difficult questions which had to be solved. When he met the men consulted he received them with courtesy and interested attention, gave the impression that his purpose was so to frame the various clauses as to make them really efficient when the Act came into working. We make bold to say that the large majority of the men who gave him most valuable help gained this impression from their interviews with Mr. Lloyd George, and that the majority of them have since been disheartened and amazed to find that, instead of making the practical working and wisdom of each clause in the Bill the first consideration, he has disregarded the advice which he was the first to ask for, and gone on his own way so far that the Act as it has been passed is universally recognised to bristle with unstatesmanlike difficulties, and has been made practically unworkable by its author's own hand. The Hospital has nothing to do with politics, but it has much to do with the health and well-being of the people, and speaking in this latter capacity we have to regret the absence of statesmanship displayed by Mr. Lloyd George and its inevitable consequences to the inhabitants of these islands.

The mischief is that under the system which prevails there at the moment all legislation, in any right meaning of the term, has become impossible in the House of Commons. Under the kangaroo closure a few men can discuss irrelevant points, taking up hours of time, so as to prevent any discussion whatever upon really important clauses which urgently need amendment in the case of a Bill of the first importance. Take what happened in regard to the relation of the hospitals to national insurance under Mr. Lloyd George's Bill. One of the foremost members in the House of Commons wrote to us in August last in reply to our inquiry as to why it was that the hospital question was not debated in Committee—"Clause 15 was passed about two o'clock in the morning. I had intended again to press the Chancellor of the Exchequer on the case of the hospitals; but after working from eight in the morning till two next morning had gone to get some food, and the clause passed quickly in a House as weary and jaded as myself, before I got back." From a gentleman who sat out the whole of the discussions in Committee on this Bill we learn that the kangaroo closure is the gravest scandal ever introduced into the House of Commons, owing to the power it gives to a few men at their will and pleasure to prevent not only adequate, but any, discussion upon particular clauses, an alteration of which in the direction of the public interest might affect adversely the private interests of those they represent. How is it possible that the National Insurance Act in such circumstances, or indeed any Bill, subject to like conditions, can ever be made a statesmanlike measure by the House of Commons under the existing procedure?

It has been a bitter disappointment that Mr. Lloyd George has so lamentably failed to exhibit the statesmanlike qualities which the country had a right to expect from him after his success at the Board of Trade. The letter to a medical correspondent which was published in the morning papers of the 10th instant eloquently proves that he is again putting what he must regard as the interests of his party before the interests of the people of these islands. The Insurance Commissioners have made no secret that it will take at least three amending Acts before the present one on national insurance can be made to work satisfactorily, if at all. We make bold to say, and we even yet hope, that the interests and welfare of the people may induce Mr. Lloyd George and the Government to reconsider their position, and to take steps to secure that a delay shall be granted in enforcing the Act pending the introduction of an amending Act removing the most glaring defects which must at present render this much desired national insurance for the British people ineffective. The question of national insurance bristled with difficulties at the commencement, but those difficulties have been multiplied rather than diminished by the absence of statesmanship and the haste with which the holder of a great office in the Cabinet has resolved to disregard the advice and impartial warnings of his advisers. Has Mr. Lloyd George grown so irresponsive and irresponsible that he is utterly callous as to the avoidable hardships and cruelties which must result to many of the 14,000,000 people whose health and lives are intimately associated with his proposals for the enforcement of national insurance? We cannot credit that this should be so, but the alternative is even worse, for it must mean a lamentable ingnorance of, and disregard for, the teachings of experience on the part of Mr. Lloyd George, which may make his name go down to history as the one Chancellor of the Exchequer who was never a statesman, and often lacked the patriotic spirit and devotion to duty in the absence of which practical legislation is impossible.

